# Patient with suspected co-infection of hemorrhagic fever with renal syndrome and malaria: a case report

**DOI:** 10.3389/fmed.2024.1341015

**Published:** 2024-05-01

**Authors:** Han-Dong Zhao, Hong-Bo Qian, Ze-Kun Wang, Rui-Kang Ren, Tong-Bo Yu, Hong-Li Liu

**Affiliations:** ^1^Central Laboratory of Virology, Shaanxi Provincial Hospital of Infectious Diseases, The Eighth Hospital Affiliated to Medical College of Xi’an Jiaotong University, Xi’an, China; ^2^Clinical Laboratory Center, Shaanxi Provincial Hospital of Infectious Diseases, The Eighth Hospital Affiliated to Medical College of Xi’an Jiaotong University, Xi’an, China; ^3^Department of Radiology, Shaanxi Provincial Hospital of Infectious Diseases, The Eighth Hospital Affiliated to Medical College of Xi’an Jiaotong University, Xi’an, China; ^4^Network and Information Center, Shaanxi Provincial Hospital of Infectious Diseases, The Eighth Hospital Affiliated to Medical College of Xi’an Jiaotong University, Xi’an, China; ^5^Clinical Laboratory Center, Xi’an People’s Hospital (Xi’an Fourth Hospital) Guang-Ren Hospital Affiliated to Xi’an Jiaotong University Health Science Center, Xi’an, China

**Keywords:** HFRS, Hantaan virus, malaria, false-positive IgM, case report

## Abstract

**Background:**

Hemorrhagic fever with renal syndrome (HFRS) is a natural epidemic disease that can be caused by the Hantaan virus (HTNV). Malaria is caused by plasmodium and can be transmitted by a mosquito bite. The similar manifestations shared by these disorders pose a challenge for clinicians in differential diagnosis, in particular, coupled with a false-positive serological test.

**Case presentation:**

A 46-year-old man was admitted for fever and chills for over 10 days and was suspected of being co-infected with HFRS and malaria due to a history of travel to malaria-endemic areas and a positive HTNV-immunoglobulin M (IgM) test. Although leukocytosis, thrombocytopenia, renal injury, lymphocytosis, overexpression of interleukin-6, and procalcitonin were observed during the hospitalization, the hypotensive, oliguria, and polyuria phases of the HFRS course were not observed. Instead, typical symptoms of malaria were found, including a progressive decrease in erythrocytes and hemoglobin levels with signs of anemia. Furthermore, because the patient had no history of exposure to HFRS endemic areas, exposure to an HTNV-infected rodent, or a positive HTNV-IgG test, and false serological tests of IgM can be caused by various factors, the HFRS coinfection with malaria was ruled out.

**Conclusion:**

Misdiagnosis can be easily induced by a false serological test, in particular the IgM test which can be influenced by various factors. A combination of health history, epidemiology, physical examination, precise application of specific examinations involving tests of conventional laboratory parameters as well as well-accepted methods such as the immunochromatographic (ICG) test, real-time reverse transcription-polymerase chain reaction (PCR), and Western blot (WB), and acquaintance with disorders with similar manifestations will contribute to the precise diagnosis in clinical treatment.

## 1 Introduction

Hemorrhagic fever with renal syndrome (HFRS), an infectious disease caused by the Hantaan virus (HTNV), has been reported in China since the first millennium and was suggested or mentioned during the American Civil War (1862–1863) and Korea, Manchuria, and Far Eastern Russia (1913–1914), respectively (1). Thereafter, the causative agent of this disorder was recognized in Korea in 1978. Annually, 60,000–150,000 cases are registered around the world, 90% of which are reported in China. The major manifestations of HFRS are characterized by fever, hemorrhage, renal impairment, and thrombocytopenia (2). Malaria, which is caused by the plasmodium, is also associated with fever, anemia, classic paroxysm, acute renal injury, and thrombocytopenia, despite the latter being uncommon (3). Actually, malaria is also an ancient human infectious disease that has been mentioned as early as 2,700 BC in Egypt and China. Approximately, 300–500 million individuals are infected by plasmodium, with a fatal number of 1.5–2.7 million every year (4). Noteworthy, considering that the disorders aforementioned have had a profound influence on socioeconomics, public health, and the medical assistance system worldwide, substantial efforts have been made to control the prevalence of these disorders, especially the application of the inactivated monovalent and bivalent vaccines for HFRS, which has greatly reduced the morbidity of this disorder in China and Korea. Meanwhile, a recombinant protein vaccine directed against the circumsporozoite protein of malaria was reported to be in phase 3 clinical trials. Nevertheless, there is no World Health Organization (WHO)-approved vaccine to prevent HTNV and plasmodium infection worldwide (3, 5). Furthermore, given the similar symptoms possessed by these two disorders, all of which pose a challenge for clinicians in differential diagnosis and may lead to delays in effective treatment.

Here, we presented a case that is characterized by the symptoms of HFRS and malaria and confused clinicians, which may eventually lead to misdiagnosis, delays in effective treatment, or even severe outcomes for the patient. Thus, this case report provides valuable further insight for clinicians in clinical practice regarding the differential/treatment of HFRS, malaria, and the coinfection of these disorders.

## 2 Case presentation

A 46-year-old man was referred to the Department of Infectious Diseases of Shaanxi Provincial Hospital of Infectious Diseases (Eighth Affiliated Hospital of the Medical College of Xi’an Jiaotong University) on 7 September 2023 with a fever for more than 10 days. Two days prior to admission, he felt fatigued, had diarrhea, and was later confirmed to be infected with plasmodium coupled with a positive of HFRS-immunoglobulin M (IgM) in another medical institution. Due to the suspicion of coinfection of HFRS with malaria, he was referred to our hospital. The patient traveled to the Republic of Cameroon but has no history of health problems or other comorbidities and has not been vaccinated for the HFRS.

**Figure 1:**
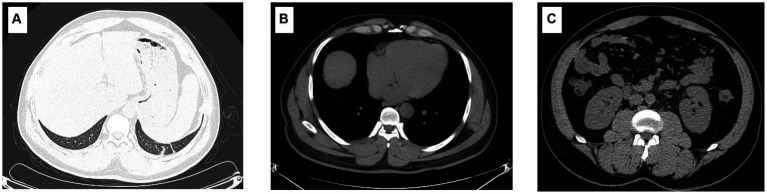
Imaging characteristics of the patient. The imaging findings show a few inflammatory cords in the lower lobe of the left lung as well as the middle lobe of the right lung **(A)**; however, the typical symptoms of HFRS, including renal edema, perirenal fascial edema, pleural effusion, and pericardial effusion, were not found **(B,C)**.

Physical examination on admission showed that body temperature, pulse rate, respiratory rate, and blood pressure were 38.6°C, 86 beats/min, 23 beats/min, and 110/66 mmHg, respectively, together with slight pharyngeal congestion. Laboratory examination during hospitalization indicated leukocytosis (peak value 13.15 × 10^9^; normal value 3.7–9.15 × 10^9^ cell/L), thrombocytopenia (nadir value 18 × 10^9^; normal value 85–303 × 10^9^ cell/L), and renal injury with an elevated level of creatine (Cr) (peak value 315.1; normal value 70–115 μmol/L), urea (peak value 27.1; normal value 1.7–8.3 mmol/L), and uric acid (UA) (peak value 684.2; normal value 204–416 μmol/L). Furthermore, overexpression of interleukin-6, procalcitonin, and B-type natriuretic peptide was also observed, which indicated inflammation and cardiovascular injury to some extent (Table 1). Besides, the abnormality of CD8^+^ T lymphocytes, B and natural killer (NK) cells were also found. Chest computer tomography (CT) scan showed a few inflammatory cords in the lower lobe of the left lung as well as the middle lobe of the right lung but with no further abnormality (Figure 1).

**Table 1:** Timeline of the disease course (7–19 September 2023).

Date (day of hospitalization)	Sep. 7 (day 1)	Sep. 8 (day 2)	Sep. 9 (day 3)	Sep. 11 (day 5)	Sep. 12 (day 6)	Sep. 13 (day 7)	Sep. 14 (day 8)	Sep. 16 (day 10)	Sep. 19 (day 13)
Leukocyte (3.7–9.15 × 10^9^/L)	9.18	9.55	9.53	13.01		13.15	11.96	11.71	6.87
Neutrophile (2–7 × 10^9^/L)	7.36	7.31	8.51	11.17		9.98	7.83	8.46	4.54
Platelet (85–303 × 10^9^/L)	84	79	68	18		53	102	201	391
Erythrocytes (3.5–5.5 × 10^12^/L)	4.94	4.62	4.57	3.95		3.12	2.98	2.75	2.48
Hemoglobin (113–151 g/L)	150	142	138	116		93	88	80	73
CD3^+^T cell (800–4,000 cell/μL)		910		1,532					824
CD4^+^T cell (410–1,590 cell/μL)		419		401					405
CD8^+^T cell (190–1,140 cell/μL)		222		1,028					326
CD4^+^CD8^+^ T cell (0–30 cell/μL)		6		5					3
B (90–600 cell/μL)		190		365					112
NK (90–590 cell/μL)		78		412					85
APTT (24–38 s)	34.4	40.4			36.75		27	29.59	38.5
PT (10–15 s)	13.49	14			14.33		10.8	11.53	12.2
Fib (2–4 g/L)	3.39	4.46			5.26		4.82	5.96	5.77
Cr (70–115 μmol/L)	9.46	130.4	114.4	218.8	315.1	296.6	242.3	178.6	138.9
Urea (1.7–8.3 mmol/L)	522.05	7.68	6.53	19.11	26.55	27.1	23.4	13.53	6.81
UA (204–416 μmol/L)		470.2	397.6	495.3	577.7	651.7	684.2	598.4	423.6
IL-6 (0–5.5 pg./mL)		25.35			15.11		7.57	20.54	
PCT (0–0.5 ng/mL)		74.53	58.32	>100		>100	96.06	15.49	2.26
BNP (≤100 ng/mL)		41.22			100.21				
ESR (mm/h)		43					150.42		
Proteinuria	Negative								
HFRS-IgM (positive/negative)		Positive		Positive				Negative	
HFRS-IgG (positive/negative)		Negative		Negative				Negative	
Malaria (positive/negative)	Positive	Positive		Positive	Positive	Positive	Negative	Negative	Negative
Typing	P.F/Ring	P.F/Ring		P.F/Trophozoite	P.F/Trophozoite	P.F/Gametophyte			
Counting (cell/μL)	4,600	45,320		4,200	4,385	1,002	0	0	0

NK, Natural killer cells; APTT, activated partial thromboplastin time; PT, prothrombin time; Fib, fibrinogen; Cr, creatinine; UA, uric acid; IL-6, interleukin 6; PCT, procalcitonin; BNP, B-type natriuretic peptide; ESR, erythrocyte sedimentation rate; P.F, Plasmodium falciparum.

**Figure 2:**
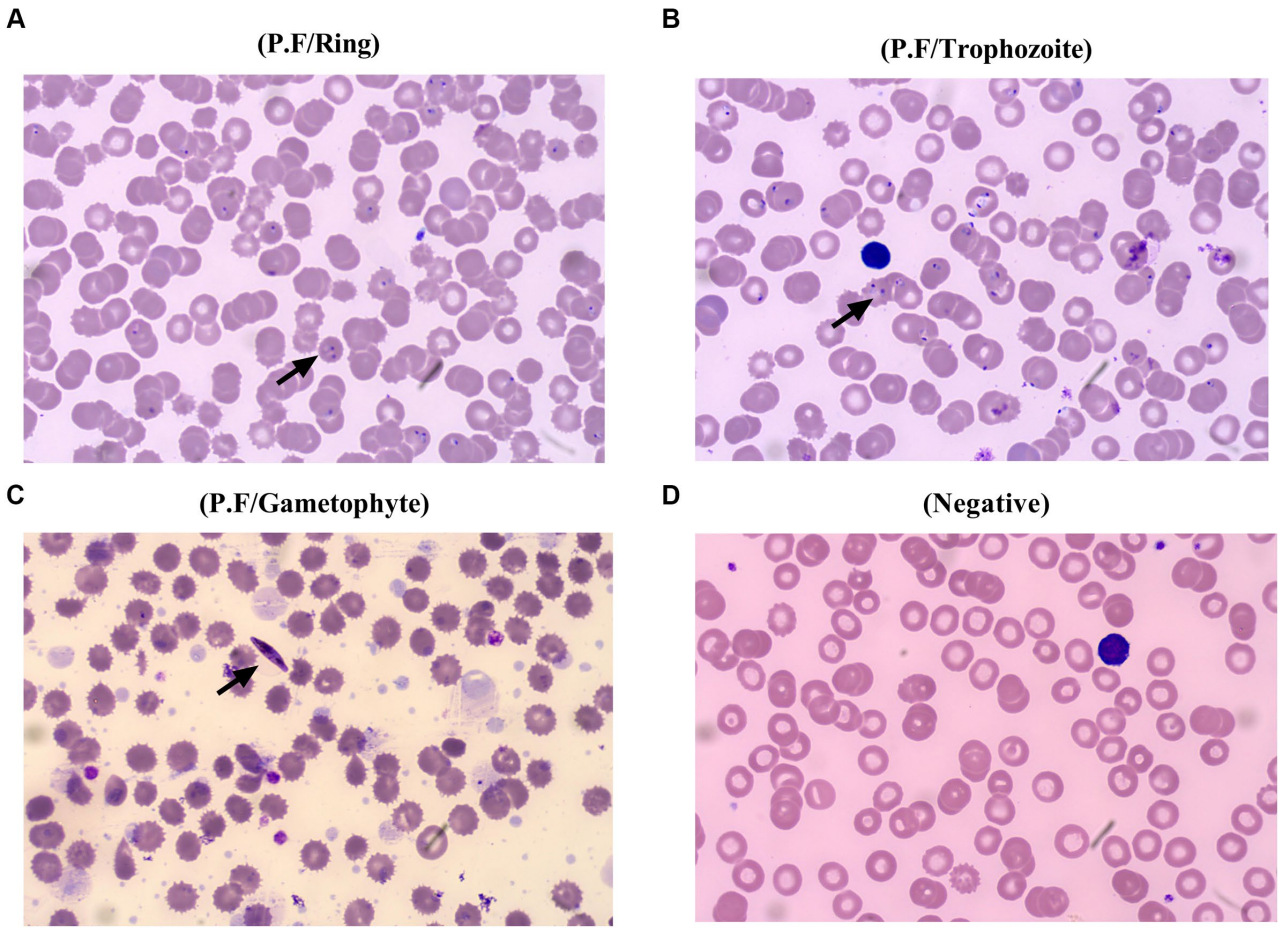
Different stages of *Plasmodium falciparum* presented in the peripheral blood of the patient. The ring of *Plasmodium falciparum* was found in the peripheral blood of the patient from the first day to the second day of admission **(A)**, and later, trophozoite of *Plasmodium falciparum* was confirmed on the fifth day of hospitalization **(B)**, followed by an emerging of gametophyte at the seventh day of hospitalization **(C)**, and plasmodium cannot be found at the 10th day of admission, which is beneficial from a precise differential diagnosis and effective specific treatment **(D)**.

Considering that the patient suffered a recurrent fever and chills coupled with confirmed malaria, while a typical manifestation of HFRS, including headache, orbital pain, back pain, abdominal pain, and blurred vision, was not found at admission. The patient was treated with artemether injection (100 mL/160 mg) for antimalaria together with supportive treatment including xiyampin (a traditional Chinese medicine, 250 mL/250 mg) for clearing heat and relieving cough, compound glycyrrhizin (a traditional Chinese medicine, 100 mL/120 mg) for hepatoprotection, and ranitidine (100 mL/50 mg) for the prevention of stress ulcers on admission. Although the number of infected red blood cells was significantly reduced on the fifth day of admission, the condition of the patient was still not stabilized, which presented as a progressing elevated level of procalcitonin, B-type natriuretic peptide, urea, and UA. Additionally, considering that the HFRS spectrum varies from the mild to the critical phases, non-specific flu-like symptoms can be detected in the early stages of HTNV-infected patients. Thus, the patient received treatment with a combination of methylprednisolone (100 mL/40 mg), ceftriaxone sodium (100 mL/2 g), and the aforementioned drugs since the sixth day of hospitalization. Nevertheless, owing to the deterioration of the disease course, which presented persistently elevated levels of C-reactive protein (CRP) (126.98 mg/L; normal value 0–10 mg/L) and leukocytes (13.15 × 10^9^; normal value 3.7–9.15 × 10^9^), the patient suffered blurred consciousness. Ceftriaxone sodium was replaced by meropenem (50 mL/0.5 g) for better anti-infection on the seventh day of hospitalization. Furthermore, artemether injection was changed to artesunate (120 mg/day) for the reason that the plasmodium-infected red blood cells still can be found under the microscope (Figure 2). Noteworthy, even though the serological test of HTNV-IgM was still positive on the fifth day of admission, the typical stages of HFRS, including febrile, hypotension, oliguria, polyuria, or HTNV-IgG, were found, while the patient presented a progressive decline of erythrocytes and hemoglobin coupled with a symptom of anemia, which coincided with the characteristics of malaria (6). Thus, the patient was treated with artesunate for antimalaria, methylprednisolone, and meropenem for anti-infection, coupled with corresponding supportive treatment as mentioned above, until the 10th day of admission.

After 13 days of hospitalization, major clinical values of the patient gradually returned to normal and were discharged from the hospital with no further discomfort. Given that his erythrocyte and hemoglobin levels were below normal, he was advised to monitor his condition and contact a clinician if he felt any discomfort.

## 3 Discussion

The world has witnessed a number of epidemics and pandemics that have taken thousands or millions of lives. Even with advances in diagnosis, monitoring, and treatment, we are still threatened by established and emerging pathogens. HFRS and malaria are among the disorders mentioned above. Although an increased effort has been made to elucidate the pathogenesis, and various measures have been applied to prevent, treat, and manage these disorders, the world has become aware of the profound influence of HFRS and malaria on public health (1, 3). Furthermore, owing to the similarities in multiple aspects shared by these disorders, it is a great challenge for clinicians to make a precise diagnosis and effective treatment timely.

The confusion in the differential diagnosis of these two disorders involves various factors, which first include similar symptoms of fever, fatigue, and thrombocytopenia. As presented herein, the patient suffered recurrent fever and a declining level of platelets with a nadir value of 18 × 10^9^/L (normal range, 85–303 × 10^9^/L), which can be both found in HFRS and malaria (1, 3), despite the fact that the mechanisms of thrombocytopenia in these two disorders may vary and remain to be elucidated. Second, the innate and adaptive immune responses involving the CD8^+^ T lymphocytes and NK cells toward HFRS and malaria are another issue that needs to be considered. As reported, immune-mediated mechanisms, in particular T cell activation in the acute phase, are associated with the pathogenesis of HFRS, and pathological damage induced by disorders of immune regulation may also contribute to the onset of HFRS (7–9). Meanwhile, a distinct change in the levels of NK cells, CD8^+^ T lymphocytes, was also observed in the HFRS patients, and the CD8^+^ T lymphocyte is considered to play a vital role in eliminating the Hantavirus (10, 11). In addition, malaria parasite-specific CD8^+^ T lymphocytes have been found in the blood of individuals living in endemic areas and after vaccination (12). Interestingly, NK cells have an influence on the generation and maintenance of productive CD8^+^ T lymphocyte responses and have also been documented to play a major role in the host response to virus infection. Besides, it has been demonstrated that NK cells contribute to the inhibition of parasite growth via the production of IFN-γ and are the first cells in peripheral blood in response to *P. falciparum* infection (13–15). Of note, abnormality of immune cells, including the CD8^+^ T lymphocytes and NK cells of the patient presented herein, was also found, which further demonstrated the significant role of immune response in the course of these two disorders, and the reversed CD4^+^ versus CD8^+^ T lymphocyte ratio observed herein, which is reported in Hantavirus-infected individuals, posed additional challenges for clinicians in differential diagnosis (16). Third, as presented in this paper, the patient suffered renal failure with an elevated level of Cr, urea, and UA; these symptoms and the corresponding mechanism are further cause for concern. As reported, the nephrotic syndrome caused by acute glomerulonephritis has been documented in patients with *P. falciparum* infection, and renal injury/failure, which is indicated by widespread capillary engorgement, focal hemorrhage, and interstitial edema, is also one hallmark of HFRS (1, 3, 17). Even though the pathogenesis is not completely understood, a large body of evidence has revealed that the endothelium is an important part of the underlying mechanisms. It has been documented that vascular endothelial cells are the primary targets of HTNV, and increasing vascular permeability is associated with various vascular adhesion molecules. Furthermore, the transformation of endothelial activation from a basic to a sticky state, which is advantageous for cell adhesion molecules binding, is considered to be an essential stage of renal injury caused by malaria (1, 3). Thus, the similarity shared by HFRS and malaria, including the symptoms, immune response, and underlying mechanism of renal injury/failure, has undoubtedly challenged the clinicians’ decision-making.

Fortunately, advances in supportive examination, particularly the development of imaging, the accumulation of clinical practice, and effective communication between the laboratory and clinical, have all made significant contributions to the differential diagnosis of various diseases. As reported herein, the patient presented a symptom of malaria coupled with a positive HTNV-IgM and was initially suspected to be a coinfection case in other medical institutions. However, because the IgM test can be influenced by various factors, including a vigorous immune response, vaccination, cross-reactivity, autoimmune diseases, heterologous reactions to similar viruses, naturally occurring biotin IgM antibodies, low pretest probability, and inappropriate test ordering, a misdiagnosis based on a single IgM test may go undetected (18). Meanwhile, the vital role of IgG in disease progression should be seriously considered because this antibody is dominant in the late phase of infection, and it also acts as a basis for ruling out the possibility of HTNV infection in this study because HTNV-IgG was never detected. Noteworthy, apart from the laboratory examination, we found that the patient had a history of travel to the Republic of Cameroon, which is an endemic area of malaria, rather than living in an endemic area of HFRS or having a history of exposure to the excreta of rodents. The HFRS infection generally occurs via the inhalation of hantavirus-contaminated aerosols from excretions, including urine, feces, and saliva (19, 20). Accordingly, the epidemiology indicated that the patient with no potential risk to HFRS infection which give us another reliable clue to get the truth. Furthermore, the patient did not present a course of HFRS such as hypotension, oliguria, and polyuria during the hospitalization but had a characteristic of malaria, including the progressed decline of erythrocytes and hemoglobin, which further demonstrated that the HTNV-IgM test is a false positive. Meanwhile, given the timepoint characteristics of HFRS prevalence, which generally emerge in winter (November to January) and early summer (May to July) (17), the patient presented herein complained of discomfort in September. Taken the information aforementioned together, the patient was preferred to be infected with malaria and treated with the corresponding antimalaria treatment. Meanwhile, the application of meropenem combined with supportive treatment in the deterioration condition reported herein greatly improved the status of the patient, which further demonstrated the advantages of this medicine in empirical therapy before the identification of causative organisms or for disease caused by single or multiple susceptible bacteria in both adults and children with a broad range of serious infections (21).

Admittedly, the case presented herein is rarely reported, and the patient was eventually getting a clear diagnosis, but there remain certain limitations. Specifically, given that this paper is a case report with limitations due to the magnitude of the study population and the absence of a control group, a large number of cases should be examined in the future to provide a powerful basis for clinicians in future clinical practice. Furthermore, since this report is a retrospective study, it posed a challenge for us to collect comprehensive and continuous medical data, even though it is inherent in case reports to some extent. Additionally, owing to the variety of methodologies such as the examination of HTNV, which can be confirmed by various methods including the enzyme-linked immunosorbent assay (ELISA), immunochromatographic (ICG) test, real-time reverse transcription-polymerase chain reaction (PCR), and Western blot (WB), as well as the variety in medicine application of the treatment, particularly the traditional Chinese medicine adopted in this study, which may not be approved or accepted out of China. Thus, the exploration of an analogous method of laboratory examination as well as medicine with similar efficacy for the disorder’s treatment is needed and would be beneficial for corresponding clinical differential diagnosis in nearing days.

## 4 Conclusion

Laboratory examinations, particularly the serological test, which is a convenient, less expensive, and time-saving method, play a key role in the supportive examinations. Nevertheless, given that the serological test can be influenced by various factors that may lead to misdiagnosis and delays in effective treatment, it is precise for clinicians to make a comprehensive decision based on a series of information collection methods involving the history of health, epidemiology, physical examination, and specific examinations that include the tests of conventional laboratory parameters and the well-accepted methods involving the ICG, real-time PCR, and WB. Considering that similar manifestations can be shared by disorders, differential diagnosis in clinical treatment not only depends on the well-acquainted characteristics of various diseases but also on the precise application of examinations.

## Data availability statement

The original contributions presented in the study are included in the article/supplementary material, further inquiries can be directed to the corresponding author.

## Ethics statement

The studies involving humans were approved by Shaanxi Provincial Hospital of Infectious Diseases (Eighth Affiliated Hospital of the Medical College of Xi’an Jiaotong University) Review Board. The studies were conducted in accordance with the local legislation and institutional requirements. The participants provided their written informed consent to participate in this study. Written informed consent was obtained from the individual(s) for the publication of any potentially identifiable images or data included in this article.

## Author contributions

H-DZ: Data curation, Resources, Writing – original draft. H-BQ: Investigation, Supervision, Writing – original draft. Z-KW: Software, Writing – original draft, Resources. R-KR: Writing – original draft, Resources. T-BY: Methodology, Software, Writing – original draft. H-LL: Funding acquisition, Writing – review & editing.
